# MicroRNA-124-3p Plays a Crucial Role in Cleft Palate Induced by Retinoic Acid

**DOI:** 10.3389/fcell.2021.621045

**Published:** 2021-06-09

**Authors:** Hiroki Yoshioka, Yurie Mikami, Sai Shankar Ramakrishnan, Akiko Suzuki, Junichi Iwata

**Affiliations:** ^1^Department of Diagnostic and Biomedical Sciences, School of Dentistry, The University of Texas Health Science Center at Houston, Houston, TX, United States; ^2^Center for Craniofacial Research, The University of Texas Health Science Center at Houston, Houston, TX, United States; ^3^MD Anderson Cancer Center UTHealth Graduate School of Biomedical Sciences, Houston, TX, United States

**Keywords:** *all-trans* retinoic acid, environmental factor, cleft palate, microRNA, craniofacial development

## Abstract

Cleft lip with/without cleft palate (CL/P) is one of the most common congenital birth defects, showing the complexity of both genetic and environmental contributions [e.g., maternal exposure to alcohol, cigarette, and retinoic acid (RA)] in humans. Recent studies suggest that epigenetic factors, including microRNAs (miRs), are altered by various environmental factors. In this study, to investigate whether and how miRs are involved in cleft palate (CP) induced by excessive intake of *all-trans* RA (*at*RA), we evaluated top 10 candidate miRs, which were selected through our bioinformatic analyses, in mouse embryonic palatal mesenchymal (MEPM) cells as well as in mouse embryos treated with *at*RA. Among them, overexpression of miR-27a-3p, miR-27b-3p, and miR-124-3p resulted in the significant reduction of cell proliferation in MEPM cells through the downregulation of CP-associated genes. Notably, we found that excessive *at*RA upregulated the expression of miR-124-3p, but not of miR-27a-3p and miR-27b-3p, in both *in vivo* and *in vitro*. Importantly, treatment with a specific inhibitor for miR-124-3p restored decreased cell proliferation through the normalization of target gene expression in *at*RA-treated MEPM cells and *at*RA-exposed mouse embryos, resulting in the rescue of CP in mice. Taken together, our results indicate that *at*RA causes CP through the induction of miR-124-3p in mice.

## Introduction

Cleft lip with/without cleft palate (CL/P) is the second most common congenital birth defect worldwide, with a prevalence of as high as 1 in 700 live births (IPDTOC Working Group, 2011). CL/P impacts on various physiological functions such as swallowing, feeding, speech, and hearing, even after multiple surgical corrections, orthodontic treatment, and speech therapy (Ferguson, 1988); therefore, the quality of life of both patients and their families is strongly diminished (Sischo et al., 2017; Kummer, 2018; De Cuyper et al., 2019). Palate development is regulated through fine-tuned spatiotemporal gene regulatory networks that control the growth, elevation, and fusion of the palatal shelves through cell migration, proliferation, apoptosis, differentiation, and extracellular matrix secretion and arrangement. Dysregulation of each step of palatogenesis results in a failure in normal palate development and causes cleft palate (CP). Our previous studies showed that at least 223 genes [a total of 198 genes related to cleft palate only (CPO), including cleft in the secondary palate, primary palate, soft palate, and submucous CP, and a total of 45 genes related to cleft lip with cleft palate (CLP), with 20 genes duplicated in CPO and CLP adjusted] in mice and 185 genes (a total of 27 genes related to CPO and a total of 177 genes related to CL/P) in humans are associated with CP (Suzuki et al., 2018a, b, 2019a). Thus, a large number of genes play crucial roles in palate development.

The etiology of CL/P is further complicated with interactions between genetic and environmental factors (Murray, 2002; Beaty et al., 2016; Gonseth et al., 2019). As for environmental factors, maternal exposures to smoking and alcohol consumption are known to be a risk for CL/P (Ericson et al., 1979; Munger et al., 1996; Romitti et al., 1999; Garland et al., 2020). In addition, several chemicals are known to be teratogens that cause CL/P [e.g., dexamethasone, dioxins, and heavy metals (Bove et al., 1995; Buser and Pohl, 2015; Suhl et al., 2018; Pi et al., 2019)]. Malnutrition and mutations in genes related to nutritional metabolic/signaling pathways are also associated with CL/P in humans and animal models. For example, retinoic acid (RA), a metabolite of vitamin A, plays important roles in cell fate determination, cellular patterning, and cell differentiation in development (Rhinn and Dolle, 2012; Roberts, 2020). Excessive RA intake causes CPO in mice (Zhang et al., 2003; Kuriyama et al., 2008; Wang et al., 2019). Mice with deletion in *Cyp26b1*, a key enzyme for RA degradation, cause CPO, micrognathia, truncation of the fore/hind limbs, and ossification defects in calvaria bones (Maclean et al., 2009). In addition, mice with a mutation in retinol dehydrogenase 10, a key enzyme for RA synthesis (*Rdh10^*m*366A*sp*^* mice), show reduced RA levels and exhibit midline cleft, syndactyly, and a malformed forebrain (Ashique et al., 2012). Mice with dominant negative mutations in retinoic acid receptor alpha (Rara^403*^) and mice with deficiency of both RA receptors alpha and gamma (*Rara^–/–^;Rarg^–/–^*) exhibit midline cleft (Damm et al., 1993; Lohnes et al., 1994; Mark et al., 1995). Thus, an appropriate amount of RA is crucial for normal embryonic development, with either too much or too low RA levels causing CP in mice. In humans, decreased serum levels of vitamin A and RA binding protein 4 (RBP4), a RA translocator, have been reported in non-syndromic CL/P patients (Zhang et al., 2014). In addition, excessive vitamin A intake is known to be associated with multiple birth defects (Lammer et al., 1985; Martinez-Frias and Salvador, 1990; Werler et al., 1990); however, the minimum teratogenic dose appears to be well above the level consumed by most women through multivitamin and vitamin A supplements during pregnancy (Mills et al., 1997; Skare et al., 2012).

A number of microRNAs (miRs), which are small non-coding RNAs (21–25 nucleotides) that regulate the expression of target genes at the post-transcriptional level (Hudder and Novak, 2008; Hou et al., 2011), play important roles in a wide array of cellular functions during the development of various tissues, including the upper lip and the palate (Shin et al., 2012; Seelan et al., 2014; Warner et al., 2014; Mukhopadhyay et al., 2019). For instance, loss of a miR-processing enzyme, such as DROSHA and DICER, results in craniofacial developmental defects in mice (Zehir et al., 2010; Nie et al., 2011; Schoen et al., 2017), and polymorphisms in *DROSHA* are associated with risk of CL/P in humans (Xu et al., 2018). In addition, mice with a deletion of miR-17-92 cluster, which is located on chromosome 14 in mice and chromosome 13 in humans, exhibit either bilateral or unilateral CLP and delayed endochondral ossification, hypoplastic lung, and cardiac ventricular septal defect (Ventura et al., 2008; de Pontual et al., 2011; Wang et al., 2013). By contrast, overexpression of *miR-17-92* in mouse palatal mesenchymal cells results in increased cell proliferation (Li et al., 2012). Thus, miRs can regulate cellular functions through the regulation of their target’s gene expression. Currently, while the importance of miRs in development is well recognized through loss-of-function studies for miRs, it remains largely unknown which miRs are elevated by environmental factors to suppress genes that are crucial for palate development.

Our previous bioinformatic studies showed that 18 miRs are possibly involved in the regulation of CP-associated genes in mice (Suzuki et al., 2018a). Recent studies show that *all-trans* RA (*at*RA) modulates miR expressions in human cancer cell lines (Liu et al., 2018, 2019). However, it is still unclear which miRs are functionally relevant in palate development and whether expression of these miRs is altered by *at*RA exposure. In this study, we investigated the mechanism of how miRs contribute to pathogenesis of CPO induced by excessive *at*RA intake.

## Materials and Methods

### Cell Culture

MEPM cells were isolated from the palatal shelves of E13.5 C57BL/6J mice, as previously described (Iwata et al., 2014). The palatal shelves from one embryo were used for each cell culture, and three independent experiments were conducted using cells from different litters. MEPM cells were maintained under Dulbecco’s modified Eagle’s medium (DMEM; Sigma Aldrich, St. Louis, MO, United States) supplemented with 10% fetal bovine serum (FBS), penicillin/streptomycin (Sigma Aldrich), 2-mercaptoethanol (Gibco, Waltham, MA, United States), and non-essential amino acids (Sigma Aldrich) at 37°C in a humidified atmosphere with 5% CO_2_.

### Animals

C57BL/6J mice were obtained from The Jackson Laboratory, Bar Harbor, ME, United States. Pregnant female mice were orally administered 40–70 mg/kg *at*RA (R2625, Sigma-Aldrich) suspended in 10% ethanol and 90% corn oil emulsion at E11.5. Control mice received an equivalent amount of emulsion without *at*RA (0.1 ml/10 g body weight). For the rescue experiments, 50 mg/kg *at*RA was orally administered at E11.5, and then the miR-124-3p inhibitor (Integrated DNA Technologies, Coralville, IA, United States) was intraperitoneally injected at 5 mg/kg at E12.5 and E13.5. The protocol was approved by the Animal Welfare Committee (AWC) and the Institutional Animal Care and Use Committee (IACUC) of UTHealth (AWC 19-0079). All mice were maintained at the animal facility of UTHealth.

### Cell Proliferation Assay

MEPM cells were plated onto 96-well cell culture plates at a density of 5,000 cells per well and treated with a mimic for negative control (4464061), miR-21a-5p (4464066; MC10206), miR-27a-3p (4464066; MC10939), miR-27b-3p (4464066; MC10750), miR-30a-5p (4464066; MC11062), miR-124-3p (4464066; MC10691), miR-141-3p (4464066; MC10860), miR-200a-3p (4464066; MC10991), miR-203-3p (4464066; MC10152), miR-320-3p (4464066; MC11621), and miR-381-3p (4464066; MC10242) [mirVana miRNA mimic (chemically modified double-stranded RNA molecules), ThermoFisher Scientific, Waltham, MA, United States], or an inhibitor for negative control (4464079), miR-27a-3p (4464084; MH10939), miR-27b-3p (4464084; MH10750), and miR-124-3p (4464084; MH10691) [mirVana miRNA inhibitor (chemically modified, single-stranded oligonucleotides with patented secondary structure), Thermo Fisher Scientific], using the Lipofectamine RNAiMAX transfection reagent (Thermo Fisher Scientific), according to the manufacturer’s protocol (3 pmol of the mimic or the inhibitor and 0.3 μl of the transfection reagent in 100 μl DMEM per well). Cell proliferation was measured using the Cell Counting Kit 8 (Dojindo Molecular Technologies Inc., Gaithersburg, MD, United States) 24, 48, or 72 h after treatments (*n* = 6 per group). For the *at*RA exposure experiments, MEPM cells were plated onto 96-well cell culture plates at a density of 5,000 cells per well and treated with 30 μM *at*RA. After 24, 48, or 72 h of treatment, cell numbers were determined as described above.

### Bromodeoxyuridine (BrdU) Incorporation Assay

MEPM cells were plated onto 35-mm dishes at a density of 25,000/dish and treated with 30 μM *at*RA or control vehicle (dimethyl sulfoxide). After 24 h, the cells were incubated with 100 μg/ml BrdU (Sigma Aldrich) for 1 h. Incorporated BrdU was stained with a rat monoclonal antibody against BrdU (ab6326; Abcam, Cambridge, MA, United States 1:1,000), as previously described (Iwata et al., 2014). A total of six fields, which were randomly selected from three independent experiments, were used for the quantification of BrdU-positive cells.

### Immunoblotting

MEPM cells were plated onto a 60-mm dish at a density of 50,000 cells per dish and treated with either 30 μM *at*RA or vehicle for 72 h, or with each miR mimic or control miR for 48 h. The treated cells were lysed with RIPA buffer (Cell Signaling Technology, Danvers, MA, United States) with a protease inhibitor cocktail (Roche, Indianapolis, IN, United States). The cells were harvested and centrifuged at 21,130 × *g* for 10 min at 4°C. The supernatant of each sample was collected, and protein concentration was determined using the BCA protein kit (Pierce). Protein samples were applied to Mini-PROTEAN TGX Gels (Bio-Rad, Hercules, CA, United States) and transferred to a polyvinylidene difluoride (PVDF) membrane. A mouse monoclonal antibody against GAPDH (MAB374, Millipore, Burlington, MA, United States, 1:6,000), a rabbit monoclonal antibody against CCND1 (2978, Cell Signaling Technology, 1:1,000), and a rabbit polyclonal antibody against cleaved caspase 3 (9661, Cell Signaling Technology, 1:1,000) were used. Peroxidase-conjugated anti-mouse IgG (7076, Cell Signaling Technology, 1:100,000) and anti-rabbit IgG (7074, Cell Signaling Technology, 1:100,000) were used as secondary antibodies.

### Quantitative RT-PCR

MEPM cells were plated onto a 60-mm dish at a density of 40,000 cells per dish. When the cells reached 80% confluence, they were treated with a mimic or an inhibitor for miR-27a-3p, miR-27b-3p, miR-124-3p, or a negative control, at 3 pmol in 6 μl of transfection reagent (Lipofectamine RNAiMAX transfection reagent in 4 ml DMEM per dish). After 24 h of treatment, total RNA was extracted with the QIAshredder and miRNeasy Mini Kit (QIAGEN, Hilden, Germany) according to the manufacturer’s instructions. For the *at*RA experiments, the cells were plated onto a 60-mm dish at a density of 50,000 cells per dish and treated with 30 μM *at*RA for 24 h, and total RNA from MEPM cells (*n* = 6 per group) was isolated as described above. For the animal experiments, palatal shelves were microdissected at E13.5 and E14.5. Total RNA (1 μg) from each sample was reverse-transcribed using iScript Reverse Transcription Supermix for qRT-PCR (Bio-Rad), and the cDNA was amplified with iTaq Universal SYBR Green Supermix (Bio-Rad) using the CFX96 Touch Real-Time PCR Detection system (Bio-Rad). The PCR primers used in this study are listed in Supplementary Table 1. The amount of each mRNA was normalized by *Gapdh*. miR expression was measured with Taqman Fast Advanced Master Mix and Taqman Advanced miR cDNA Synthesis Kit (Thermo Fisher Scientific), according to the manufacturer’s instructions. Probes for miR-27a-3p (mmu478384_mir), miR-27b-3p (mmu478270_mir), miR-124-3p (mmu480901_mir), and miR-26a-5p (477995_mir) were purchased from Thermo Fisher Scientific.

### Histological Analysis

The embryos’ heads were collected at E13.5, E14.5, and E18.5 and fixed with 4% paraformaldehyde overnight. After decalcification with 10% ethylenediaminetetraacetic acid-2Na-2H_2_O (EDTA), all samples were dehydrated and embedded in paraffin. Paraffin-embedded tissues were sectioned at 4-μm thickness and stained with hematoxylin and eosin (H&E). For immunohistochemistry, paraffin sections were deparaffinized and rehydrated. After antigen retrieval treatment with citrate buffer (pH 6.0) and blocking of endogenous peroxidase with 0.3% hydrogen peroxide in methanol, the sections were incubated with anti-cytokeratin 14 mouse monoclonal antibody (Abcam, ab7800, 1:200 dilution), anti-Ki-67 rabbit monoclonal antibody (Abcam, ab16667, 1:200 dilution), anti-VCAN rabbit polyclonal antibody (Novus Biologicals, Centennial, CO, United States, NBP1-85432, 1:200 dilution), or anti-CDC42 rabbit polyclonal antibody (Proteintech, Rosemont, IL, United States, 10155-1-AP, 1:50 dilution) at 4°C overnight. The sections were then incubated with a secondary antibody, goat anti-rabbit IgG-Alexa Fluor 488 (Thermo Fisher Scientific; A-11008; 1:500 dilution) or goat anti-rabbit IgG (H + L), biotinylated (Vector Laboratories, Burlingame, CA, United States; BA-1000; 1:500 dilution) for 1 h at room temperature. Sections were counterstained with 4′,6-diamidino-2-phenylindole (DAPI) for nuclear staining for fluorescent imaging and methylene blue for bright field imaging. Azan staining was performed as previously described (Iwata et al., 2013). A total of six fields, which were randomly selected from three independent experiments, were used for the quantification of Ki-67-positive cells. Fluorescence images were obtained using a confocal microscope (Ti-C2, Nikon, Melville, NY, United States), and color images were obtained using a light microscope (BX43, Olympus, Center Valley, PA, United States); *n* = 6 per group in each experiment.

### Craniofacial Tissue Explant Culture

Timed-pregnant mice were euthanized at E13.5 and decapitated in PBS. The mandible and tongue were removed from the embryos, and each explant, including the upper half of the head, was placed in a glass tube containing BGJb medium (Gibco, 12591) supplemented with 50% fetal bovine serum, 0.1% ascorbic acid, and antibiotics. The tubes were placed in a rotary apparatus rotating at 50 rpm in an incubator at 37°C and 5% CO_2_. After 3 days in culture with/without 30 μM *at*RA, the explants were fixed in 4% PFA and processed.

### Flow Cytometry

MEPM cells were plated onto 60-mm dishes at a density of 50,000/dish and treated with 30 μM *at*RA or control vehicle (dimethyl sulfoxide). After 24 h, the cells were harvested by trypsin and washed twice with cold BioLegend’s Cell Staining Buffer (BioLegend, San Diego, CA, United States; 420201). Cells were centrifuged at 500 g for 5 min at 4°C. The cell pellets were resuspended with Annexin V Binding Buffer (BioLegend; 422201) at a concentration of 1.5 × 10^6^ cells/ml. Resuspended cells (100 μl) were transferred into a Falcon tube and incubated with 5 μl of Annexin V (BioLegend: 421301) and 10 μl of propidium iodide (BioLegend: 421301) for 15 min at room temperature in the dark. A volume of 400 μl of Annexin V Binding Buffer was added to the Falcon tube, and the samples were analyzed with FACS Aria II (BD Biosciences, San Jose, CA, United States).

### Statistical Analysis

All experiments were performed independently three times. All statistical analyses were performed using the SPSS software (version 26.0, IBM, Armonk, NY, United States). The statistical significance of the differences between two groups (control and treated groups) was evaluated using independent *t*-tests. The statistical significance for multiple two groups was evaluated using multiple *t*-tests after Bonferroni correction. An adjusted *p*-value after Bonferroni correction (equivalent to non-adjusted *p* < 0.05) was considered to be statistically significant. For a comparison among multiple groups (e.g., control, treated, and rescued groups) with one factor such as gene or positive cell, a one-way analysis of variance (ANOVA) with Tukey’s honest significant difference test was used for assessment. For a comparison among multiple groups (e.g., control, treated, and rescued groups) with multiple factors, a two-way ANOVA with Tukey’s honest significant difference test was used for assessment. Cell proliferation assays were analyzed using a two-way ANOVA with Dunnett’s (vs. control) or Tukey’s (between all groups) honest significant difference test. A *p* < 0.05 was considered to be statistically significant. Data are represented as mean ± standard deviation in the graphs.

## Results

### Overexpression of miR-27a-3p, miR-27b-3p, and miR-124-3p Inhibits Cell Proliferation in MEPM Cells

**FIGURE 1:**
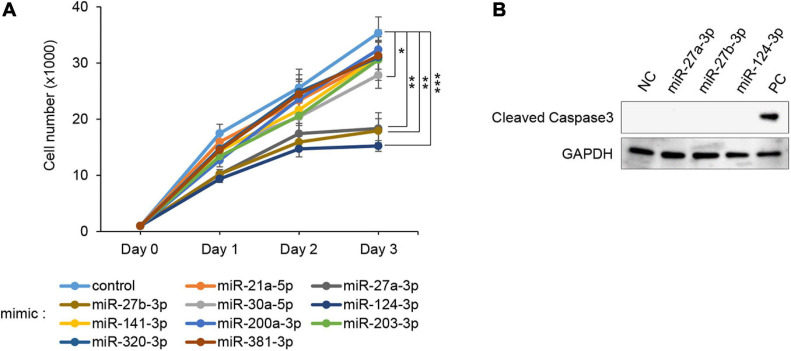
Effect of overexpression of the predicted miRs on cell proliferation in MEPM cells. **(A)** Cell proliferation assays using MEPM cells from E13.5 palatal shelves treated with the indicated miR mimic; control, miR-21a-5p, miR-27a-3p, miR-27b-3p, miR-30a-5p, miR-124-3p, miR-141-3p, miR-200a-3p, miR-203-3p, miR-320, and miR-381-3p mimic. Two-way ANOVA with Dunnett’s test (*n* = 6). *p < 0.05, **p < 0.01, ***p < 0.001. Each treatment group was compared with the control. **(B)** Immunoblotting analysis for cleaved caspase 3 in MEPM cells treated with control, miR-27a-3p, miR-27b-3p, or miR-124-3p mimic for 48 h. Intestine was used as a positive control (PC). GAPDH was used as an internal control. Representative images from two independent experiments are shown.

**FIGURE 2:**
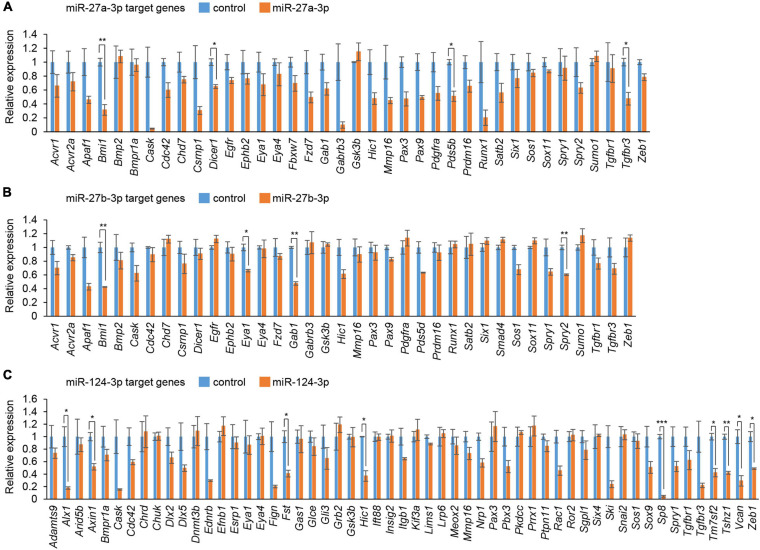
Cleft palate-associated genes suppressed by overexpression of miR-27a-3p, miR-27b-3p, or miR-124-3p in MEPM cells. **(A–C)** Quantitative RT-PCR for the indicated genes after treatment with control or miR-27a-3p mimic **(A)**, control or miR-27b-3p mimic **(B)**, and control or miR-124a-3p mimic **(C)**. Multiple *t*-tests adjusted by Bonferroni (*n* = 3). *Adjusted *p* < 0.00132 in A (38 genes), adjusted *p* < 0.00135 in B (37 genes), adjusted *p* < 0.000909 in C (55 genes), **adjusted *p* < 0.000263 in A (38 genes), adjusted *p* < 0.000270 in B (37 genes), adjusted *p* < 0.000182 in C (55 genes), ***adjusted *p* < 0.0000182 in C (55 genes).

To evaluate the effect of overexpression of miRs, which were predicted through our bioinformatic analyses (Suzuki et al., 2018a,b), MEPM cells were treated with each miR mimic (miR-21a-5p, miR-27a-3p, miR-27b-3p, miR-30a-5p, miR-124-3p, miR-141-3p, miR-200a-3p, miR-203-3p, miR-320-3p, and miR-381-3p) and analyzed for their effect on cell proliferation. Among them, miR-27a-3p, miR-27b-3p, miR-30a-5p, and miR-124-3p mimics significantly inhibited cell proliferation in MEPM cells, while the mimic of either miR-21a-5p, miR-141-3p, miR-200a-3p, miR-203-3p, miR-320-3p, or miR-381-3p did not affect cell proliferation (Figure 1A). We confirmed that miR-27a-3p, miR-27b-3p, and miR-124-3p mimics did not induce apoptosis (Figure 1B). To identify CP-associated genes targeted by either the miR-27a-3p, miR-27b-3p, or miR-124-3p mimic, we conducted quantitative RT-PCR analysis for the predicted target genes (38 CP-associated genes in miR-27a-3p, 37 CP-associated genes in miR-27b-3p, and 55 CP-associated genes in miR-124-3p) in MEPM cells after treatment with each miR mimic (Supplementary Table 2). Among them, the expression of four genes (*Bmi1*, *Dicer1*, *Pds5b*, and *Tgfbr3*) in the miR-27a-3p mimic, four genes (*Bmi1*, *Eya1*, *Gab1*, and *Spry2*) in the miR-27b-3p mimic, and nine genes (*Alx1*, *Axin1*, *Fst*, *Hic1*, *Sp8*, *Tm7sf2*, *Tshz1*, *Vcan*, and *Zeb1*) in the miR-124-3p mimic were significantly downregulated in candidate target genes downregulated with treatment of each miR mimic (Figures 2A–C). To further evaluate the role of each miR in cell proliferation and gene regulation, we treated MEPM cells with a specific inhibitor for either miR-27a-3p, miR-27b-3p, or miR-124-3p. We found that inhibitors of miR-27a-3p, miR-27b-3p, and miR-124-3p failed to change cell proliferation activity (Supplementary Figure 1A). We then performed quantitative RT-PCR analysis for the predicted target genes and found that the expression of 17 genes (*Acvr2a*, *Bmi1*, *Cdc42*, *Chd7*, *Ephb2*, *Eya4*, *Gab1*, *Pax9*, *Pdgfra*, *Prdm16*, *Runx1*, *Six1*, *Sox11*, *Spry1*, *Spry2*, *Tgfbr3*, and *Zeb1*) in the miR-27a-3p inhibitor, 6 genes (*Apaf1*, *Eya4*, *Pds5d*, *Sos1*, *Spry2*, and *Sumo1*) in the miR-27b-3p inhibitor, and 9 genes (*Axin1*, *Cdc42*, *Esrp1*, *Fst*, *Gas1*, *Mmp16*, *Pbx3*, *Vcan*, and *Zeb1*) in the miR-124-3p inhibitor were significantly upregulated in candidate target genes upregulated with treatment of each miR inhibitor (Supplementary Figures 1B–D). Therefore, these results suggest that *Bmi1* and *Tgfbr3* in miR-27a-3p, *Spry2* in miR-27b-3p, and *Axin1*, *Fst*, *Vcan*, and *Zeb1* in miR-124-3p were strong candidates regulated by the miRs in a dose-dependent manner.

### *at*RA Induces miR-124-3p Expression in MEPM Cells

**FIGURE 3:**
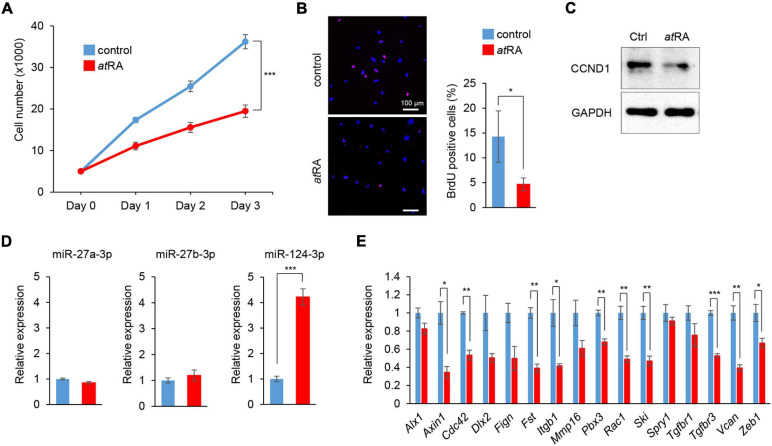
Influence of *at*RA treatment on cell proliferation and gene expression in MEPM cells. **(A)** Cell proliferation assays in MEPM cells treated with 30 μM *at*RA for 24, 48, and 72 h. Two-way ANOVA with Dunnett’s test (*n* = 6). ***p < 0.001 vs. control. **(B)** BrdU staining (red) in MEPM cells after treatment with 30 μM *at*RA for 72 h. Nuclei were counterstained with DAPI (blue). Representative images from two independent experiments are shown. Graph shows the quantification of BrdU-positive cells. Independent *t*-test. *p < 0.05 vs. control. Bars, 50 μm. **(C)** Immunoblotting analysis of CCND1 and GAPDH in MEPM cells treated with 30 μM *at*RA for 72 h. Representative images from two independent experiments are shown. **(C)** Immunoblotting analysis for CCND1 in MEPM cells treated with 30 μM *at*RA for 72 h. GAPDH was used as an internal control. **(D)** Quantitative RT-PCR for miR-27a-3p, miR-27b-3p, or miR-124-3p after treatment with *at*RA for 24 h in MEPM cells. Multiple *t*-tests adjusted by Bonferroni (*n* = 3). ***Adjusted *p* < 0.00033 (three miRs). **(E)** Quantitative RT-PCR for the indicated genes after treatment with *at*RA for 24 h in MEPM cells. Multiple *t*-tests adjusted by Bonferroni (*n* = 3–6). *Adjusted *p* < 0.00313 (16 genes), **adjusted *p* < 0.000625 (16 genes), ***adjusted *p* < 0.0000625 (16 genes) vs. control.

We conducted cell proliferation assays using MEPM cells treated with *at*RA. To determine the *at*RA concentration that affects cell proliferation, the cells were treated with 10 and 30 μM *at*RA. We found that 30 μM *at*RA significantly inhibited cell proliferation in MEPM cells (Figure 3A and Supplementary Figure 2A). The reduced cell proliferation by *at*RA was confirmed with BrdU incorporation assays (Figure 3B) and expression of Cyclin D1 (CCND1), a cell cycle accelerator, in MEPM cells treated with *at*RA (Figure 3C). In addition, cleaved caspase 3, an indicator of apoptosis, was undetectable in cells treated and untreated with *at*RA (Supplementary Figure 2B). Moreover, flow cytometry analysis showed no significant change in the profile (cell death vs. healthy cells) of cells treated with *at*RA (Supplementary Figure 2C). Taken together, these data indicate that *at*RA inhibits the proliferation of MEPM cells. Interestingly, the expression of miR-124-3p was specifically induced by *at*RA treatment (Figure 3D); by contrast, expression of miR-27a-3p and miR-27b-3p was not altered by *at*RA treatment. As expected, the miR-124-3p target genes (*Axin1*, *Fst*, *Vcan*, and *Zeb1*) were significantly downregulated with *at*RA treatment (Figure 3E). Taken together, these observations suggest that the expression of these genes was downregulated by *at*RA through miR-124-3p. We confirmed that the expression of all the other predicted genes regulated by miR-124-3p (additional 39 genes) was not correlated with the *at*RA condition (Supplementary Figure 3). For instance, although three genes (*Bmpr1a*, *Gli3*, and *Snal2*) were significantly downregulated and *Six4* was significantly upregulated under *at*RA treatment, the expression of these genes was not altered with treatment with the miR-124-3p mimic. Therefore, the use of both a mimic and an inhibitor for the identification of genes regulated by miR-124-3p was helpful to identify genes directly regulated by miR-124-3p.

### miR-124-3p Inhibitor Partially Rescues Decreased Cell Proliferation in *at*RA-Treated Cells

**FIGURE 4:**
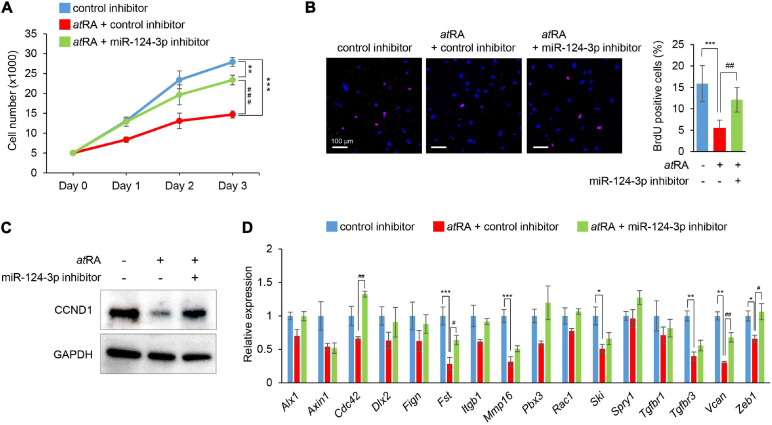
Normalization of *miR-124-3p* expression restores *at*RA-induced cell proliferation defect in MEPM cells. **(A)** Cell proliferation assays in MEPM cells treated with a control or a miR-124-3p inhibitor under the 30 μM *at*RA condition for 0, 24, 48, or 72 h (Days 0–3). Two-way ANOVA with Tukey’s honest significant difference test (*n* = 6 per group). **p < 0.01, ***p < 0.001, ^###^*p* < 0.001. **(B)** BrdU staining (red) in MEPM cells after treatment with/without a miR-124-3p inhibitor under the 30 μM *at*RA condition for 72 h. Nuclei were counterstained with DAPI (blue). Representative images from two independent experiments are shown. Graph shows the quantification of BrdU-positive cells. One-way ANOVA with Tukey’s honest significant difference test. ***p < 0.001 vs. control inhibitor + vehicle, ^##^*p* < 0.01 vs. control inhibitor + *at*RA. Bars, 50 μm. **(C)** Immunoblotting analysis of CCND1 and GAPDH in MEPM cells treated with 30 μM *at*RA under the effect of a miR-124-3p inhibitor for 72 h. Representative images from two independent experiments are shown. **(D)** Quantitative RT-PCR for the indicated genes in MEPM cells after treatment with *at*RA under the effect of a miR-124-3p inhibitor for 24 h. *Adjusted *p* < 0.00313 (16 genes), **adjusted *p* < 0.000625 (16 genes), and ***adjusted *p* < 0.0000625 (16 genes) vs. control inhibitor + vehicle. ^#^Adjusted *p* < 0.00313 (16 genes), ^##^adjusted *p* < 0.000625 (16 genes), and ^###^adjusted *p* < 0.0000625 (16 genes). Two-way ANOVA with Tukey’s honest significant difference test (*n* = 3–6).

To evaluate the contribution of miR-124-3p to *at*RA-induced cell proliferation inhibition, we treated MEPM cells with a miR-124-3p inhibitor under *at*RA treatment. The miR-124-3p inhibitor specifically suppressed miR-124-3p expression for treatment at 24 and 48 h (Supplementary Figures 4A,B). The miR-124-3p inhibitor could partially rescue reduced cell proliferation (Figure 4A). As expected, both the number of BrdU-positive cells and CCND1 expression were normalized with treatment with the miR-124-3p inhibitor (Figures 4B,C). In addition, the expression of miR-124-3p target genes (*Fst*, *Vcan*, and *Zeb1*) was partially normalized with the miR-124-3p inhibitor under *at*RA conditions (Figure 4D). Taken together, our results indicate that *at*RA inhibits cell proliferation through miR-124-3p expression in MEPM cells.

### *at*RA Induces miR-124-3p Expression in the Developing Palate in Mice

**FIGURE 5:**
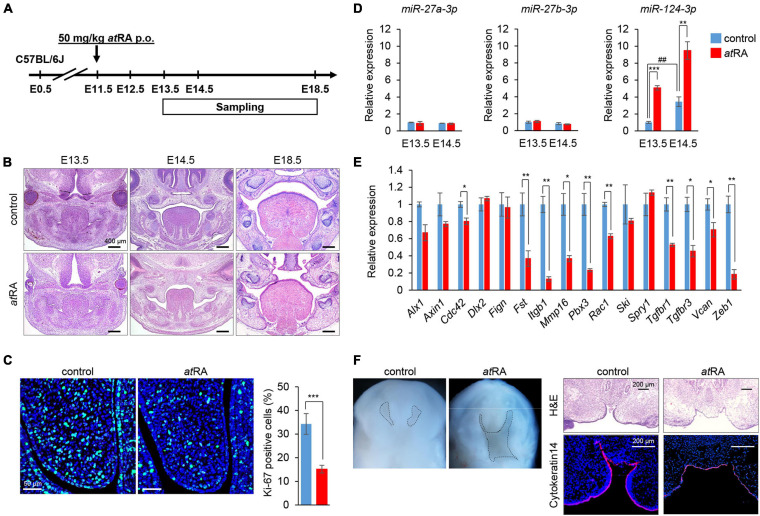
Influence of *at*RA administration on palatal development and gene expression in mice. **(A)** Schematic experimental design. **(B)** H&E staining of face at E13.5, E14.5, and E18.5. Bars, 400 μm. **(C)** Ki-67 staining (green) in the developing palate of E13.5 mice treated with control vehicle or *at*RA (50 mg/kg p.o. at E11.5). Nuclei were counterstained with DAPI (blue). Representative images from two independent experiments are shown. Graph shows the quantification of Ki-67-positive cells. Independent *t*-tests. ***p < 0.001 vs. control. Bars, 50 μm. **(D)** Quantitative RT-PCR for miR-27a-3p, miR-27b-3p, or miR-124-3p after administration of *at*RA in palatal shelves at E13.5 and E14.5. Two-way ANOVA with Tukey’s honest significant difference test (*n* = 3). **p < 0.01, ***p < 0.001 vs. control at each indicated day and ^##^*p* < 0.01 vs. E13.5 control. **(E)** Quantitative RT-PCR for the indicated genes after treatment with *at*RA for 24 h in palatal shelves at E14.5. Multiple *t*-tests adjusted by Bonferroni (*n* = 3–6). *Adjusted *p* < 0.00313 (16 genes), **adjusted *p* < 0.000625 (16 genes) vs. control. **(F)** Intraoral views of heads (left panels) from E13.5 embryos, from which the mandible and tongue were removed and cultured for 3 days with control vehicle or *at*RA (*n* = 4 per group). Dashed lines delineate the palatal shelves. H&E staining and K14 immunostaining (red) (right panels) of coronal sections of 3-day cultured explants with control vehicle or *at*RA. The nuclei were counterstained with DAPI (blue). Bars, 200 μm.

The oral administration of *at*RA to pregnant mice is known to induce CP (Abbott et al., 1989; Ross et al., 2000; Wang et al., 2002; Lai et al., 2003; Yao et al., 2011; Havasi et al., 2013; Hu et al., 2013; Hou et al., 2019; Roberts, 2020). However, the amount and timing of *at*RA administration that induces CP may differ in each mouse strain and protocol. Therefore, we tested various doses (40, 50, 60, and 70 mg/kg) of oral *at*RA in C57BL/6J mice. We found that *at*RA administration at 40 mg/kg induced CP with 57% penetrance (12/21 embryos), while more than 50 mg/kg *at*RA induced CP with 100% penetrance (Table 1). Therefore, we administered a single dose of 50 mg/kg *at*RA at E11.5 in this study (Figure 5A). The palatal shelves of embryos treated with vehicle were normally elevated and fused at E14.5, while the palatal shelves of embryos treated with *at*RA failed to elevate at E14.5, causing CP at E18.5 (Figure 5B). To confirm the reduction of cell proliferation in the developing palates of mice treated with *at*RA, we evaluated cell proliferation by Ki-67 immunostaining. As expected, the number of Ki-67-positive cells (i.e., proliferating cells) was significantly reduced in the palatal shelves of *at*RA-treated embryos (Figure 5C). Next, we microdissected the palatal shelves at E13.5 and E14.5 from mice treated with either vehicle or *at*RA and measured the miR expression. In the vehicle control group, the expression of miR-27a-3p and miR-27b-3p was not changed between E13.5 and E14.5, while the expression of miR-124-3p at E14.5 was upregulated compared to that of E13.5 (Figure 5D). The expression of miR-124-3p was significantly upregulated with *at*RA administration at both E13.5 and E14.5 compared to controls, while the expression of miR-27a-3p and miR-27b-3p was comparable to the controls (Figure 5D). Furthermore, quantitative RT-PCR analysis for genes targeted by miR-124-3p confirmed that a total of three genes (*Fst*, *Vcan*, and *Zeb1*) were significantly downregulated in the palatal shelves of mice given *at*RA compared to controls (Figure 5E). To confirm that the hypoplastic mandible secondarily caused CP in mice treated with *at*RA, we cultured E13.5 craniofacial explants, which were extracted from the mandible and tongue, with/without *at*RA for 3 days (Figure 5F). In controls, the palatal shelves were elevated and almost completely fused. By contrast, the explants treated with *at*RA showed a widely opened palate. These results indicate that *at*RA primarily causes CP in mice treated with *at*RA.

**TABLE 1:** Incidence of cleft palate (CP) by *at*RA administration at different doses.

***at*RA dose (mg/kg)**	**Total incidence of CP**	**Percentage of CP**
40	12/21	57%
50	14/14	100%
60	21/21	100%
70	34/34	100%

### Normalization of miR-124-3p Expression Can Rescue CP Induced by *at*RA in Mice

**FIGURE 6:**
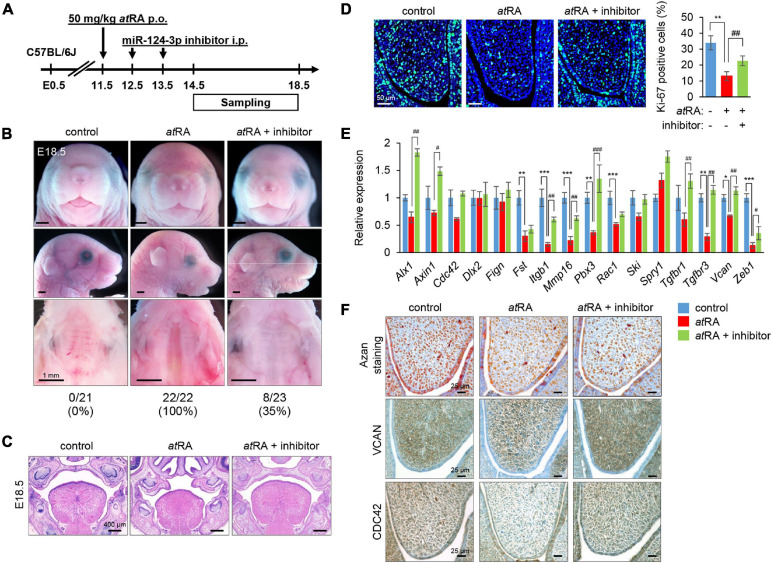
Inhibition of miR-124-3p expression rescues *at*RA-induced CP in mice. **(A)** Schematic experimental design. **(B)** Gross picture of E18.5 C57BL/6J mice treated with control vehicle, *at*RA, or *at*RA + miR-124-3p inhibitor. Bars, 1 mm. **(C)** H&E staining of the face of E18.5 C57BL/6J mice treated with control vehicle, *at*RA, or *at*RA + miR-124-3p inhibitor. Bars, 400 μm. **(D)** Ki-67 staining (green) in the developing palate of E13.5 C57BL/6J mice treated with/without a miR-124-3p inhibitor under *at*RA condition. Nuclei were counterstained with DAPI (blue). Representative images from two independent experiments are shown. Graph shows the quantification of Ki-67 positive cells. One-way ANOVA with Tukey’s honest significant difference test. **p < 0.01 vs. control. ^##^*p* < 0.01 vs. *at*RA. Bars, 50 μm. **(E)** Quantitative RT-PCR for the indicated genes after treatment with/without a miR-124-3p inhibitor under *at*RA condition in the palatal shelves of E14.5 C57BL/6J mice. Two-way ANOVA with Tukey’s honest significant difference test (*n* = 3). *Adjusted *p* < 0.00313 (16 genes), **adjusted *p* < 0.000625 (16 genes), and ***adjusted *p* < 0.0000625 (16 genes) vs. control. ^#^Adjusted *p* < 0.00313 (16 genes), ^##^adjusted *p* < 0.000625 (16 genes), and ^###^adjusted *p* < 0.0000625 (16 genes) vs. *at*RA. **(F)** AZAN staining (upper panels) and immunohistochemical staining for VCAN (middle panels; brown) and CDC42 (bottom panels; brown) in E13.5 C57BL/6J mice treated with control vehicle, *at*RA, or *at*RA + miR-124-3p inhibitor. Bars, 25 μm.

Finally, we attempted to rescue CP induced by *at*RA in mice through treatment with an inhibitor specific for miR-124-3p (Figure 6A). The administration of a miR-124-3p inhibitor reduced CP penetrance from 100 to 35% in the *at*RA-induced CPO mouse model (Figures 6B,C). The reduced cell proliferation was partially normalized in mice injected with a miR-124-3p inhibitor under *at*RA-treated conditions (Figure 6D). Moreover, the expression of *Vcan* and *Zeb1* was partially normalized with a miR-124-3p inhibitor upon *at*RA exposure (Figure 6E). To analyze the expression pattern of extracellular matrix (ECM) formation in mice treated with *at*RA, we performed Azan staining for the visualization of overall ECM production and pattern. We did not detect any alteration in ECM patterning. Furthermore, to confirm that there was alteration in the patterning, but not in the level, of expression of the molecules, we conducted immunohistochemical analyses for VCAN and CDC42 in mice treated with *at*RA and confirmed that there was no change in the expression pattern (Figure 6F). Taken together, our results indicate that *at*RA induces CPO through the upregulation of miR-124-3p expression in mice.

## Discussion

miRs have been implicated in developmental processes and various types of cancers (Mukhopadhyay et al., 2010; Chen et al., 2012), and dysregulation of miR expression has been identified in a variety of human diseases (Mendell and Olson, 2012). Recent studies show that miRs play important roles in craniofacial development (Shin et al., 2012; Seelan et al., 2014; Warner et al., 2014; Mukhopadhyay et al., 2019); for example, administration of miR-23b and miR-133 duplex results in facial defects in zebrafish (Ding et al., 2016). A bioinformatic study suggested that miR-199a-5p may be associated with CL/P through the modulation of transforming growth factor (TGF)-α expression (Chen et al., 2018). miR-140-5p overexpression inhibits cell proliferation through the suppression of genes associated with CP, *Fgf9*, and *Pdgfra* in MEPM cells (Li et al., 2020). Mice with a deletion of the miR-17-92 cluster (includes six miRs: miR-17, miR-18a, miR-19a, miR-19b-1, miR-20a, and miR-92a-1) exhibit CP through altered bone morphogenetic protein (BMP) signaling (Wang et al., 2013).

In this study, we found that the overexpression of miR-27a-3p, miR-27b-3p, and miR-124-3p could inhibit cell proliferation through the suppression of genes associated with mouse CP. miR-27 is a small somatic-enriched miR family comprising two paralogous members, miR-27a and miR-27b, in vertebrates (Kozomara and Griffiths-Jones, 2014). In zebrafish, miR-27 is highly expressed in the pharyngeal arches, and knockdown of miR-27 results in a complete loss of pharyngeal cartilage due to downregulated cell proliferation (Kara et al., 2017). miR-27a-3p, a member of the miR-23a/27a/24-2 cluster, is significantly upregulated in laryngeal carcinoma (Tian et al., 2014) and plays important roles in cell proliferation and migration in human gastric and breast cancers (Mertens-Talcott et al., 2007; Zhou et al., 2016). miR-27b-3p, a member of the miR-23b/27b/24-1 cluster, promotes cell proliferation and invasion in breast cancers (Jin et al., 2013), while miR-27b-3p inhibits cell growth and tumor progression in neuroblastoma (Lee et al., 2012), suggesting that miR-27b-3p functions are dependent on cancer types. In addition, a recent study showed that circulating miR-27b might be a potential biomarker for non-alcoholic fatty liver disease (Tan et al., 2014), and another study showed that miR-27b-3p is associated with CP in non-syndromic CL/P (Wang et al., 2017).

miR-124-3p is abundant in the human and mouse brain (Lagos-Quintana et al., 2002; Landgraf et al., 2007). In addition, miR-124-3p acts as a tumor suppressor against breast cancer and hepatocellular carcinoma (Liang et al., 2013; Long et al., 2018). Our previous study showed that the overexpression of miR-124-3p suppresses cell proliferation in primary mouse lip mesenchymal cells (Suzuki et al., 2019b). miR-124-3p regulates cell cycle by targeting integrin subunit beta 1 (ITGB1) in oral squamous cell carcinoma cells (Hunt et al., 2011), suggesting that miR-124-3p expression might remain at lower levels during normal palate development. Therefore, miR-124-3p may be a potential biomarker and a therapeutic target to prevent and diagnose CP.

In this study, we found that *Bmi1* and *Tgfbr3* in miR-27a-3p, *Spry2* in miR-27b-3p, and *Axin1*, *Fst*, *Vcan*, and *Zeb1* in miR-124-3p were regulated in MEPM cells through both gain-of-function and loss-of-function assays for miR in gene regulation. Because these genes are involved in various signaling pathways, including Wnt/β-catenin, BMP, TGF-β, and epidermal growth factor (EGF) signaling (Iwata et al., 2011; Reynolds et al., 2020), the overexpression of these miRs might lead to the suppression of multiple CP-associated genes through the dysregulation of these signaling pathways. In addition, we found that *at*RA specifically induced miR-124-3p, which suppressed the expression of genes associated with CP. While the expression of some predicted genes (*Dlx2*, *Fign*, *Ski*, *Spry1*, *Tgfbr1*, and *Vcan*) targeted by miR-124-3p was not changed by *atRA* in MEPM cells and mouse palatal shelves, these genes may be regulated by a combination of other miRs or through feedback loops. In addition, since four genes (*Axin1*, *Fst*, *Vcan*, and *Zeb1*) were downregulated in both *miR-124-3p* overexpression and *at*RA treatment conditions, these genes may be closely associated with *at*RA-induced CP in mice. AXIN1 (a WNT signaling negative regulator), FST (a BMP signaling inhibitor), and VCAN (an extracellular matrix) regulate growth factor signaling, which plays crucial roles in embryonic development; therefore, the fine-tuning regulation of the signaling pathways is important for proper embryogenesis. Homeobox gene ZEB1 regulates the epithelial-mesenchymal transition (EMT) in cancer and embryonic development (Schmalhofer et al., 2009; Gheldof et al., 2012); miR-200 family plays a role in EMT by regulating the *ZEB1* expression in the developing palate in mice (Carpinelli et al., 2020).

Our rescue experiments show that a miR-124-3p inhibitor can reduce CP penetrance in the *at*RA-induced CP mouse model. Thus, our study is useful to identify the causative molecular mechanism of CP. However, there are some limitations in this study. First, it is unclear whether any combination of miR inhibitors can achieve better outcomes. In a future study, we will investigate which miRs are the most significantly associated with *at*RA-induced CP. Second, the expression of most genes was tested only by quantitative RT-PCR analysis. A future study may include not only the level but also the patterning of gene expression associated with *at*RA-induced CP. Finally, *at*RA is known to induce tongue abnormalities (Cong et al., 2014), and this study did not exclude the possibility that *at*RA-induced CP was secondarily caused by tongue abnormalities. A future study will test whether or not tongue anomalies due to *at*RA administration cause CP using a tissue explant culture system. Despite these limitations, the understanding of miR dysregulation by *at*RA will shed light on the link between environmental and genetic factors in CP.

## Data Availability Statement

The original contributions presented in the study are included in the article/Supplementary Material, further inquiries can be directed to the corresponding author/s.

## Ethics Statement

The animal study was reviewed and approved by the Animal Welfare Committee (AWC) and the Institutional Animal Care and Use Committee (IACUC) of UTHealth (AWC19-0079).

## Author Contributions

AS and JI designed the research. HY, YM, and SR performed the experiments. HY, AS, and JI wrote the manuscript. All authors reviewed the results and approved the final version of the manuscript.

## Conflict of Interest

The authors declare that the research was conducted in the absence of any commercial or financial relationships that could be construed as a potential conflict of interest.
