# Supplementary material for: MicroRNA-124-3p Plays a Crucial Role in Cleft Palate Induced by Retinoic Acid
Source: Front Cell Dev Biol. 2021 Jun 9;9:621045. doi: 10.3389/fcell.2021.621045 (PMC8219963; doi:10.3389/fcell.2021.621045)

Supplementary Information

Figure legends

Supplementary Figure S1. Effect of inhibition of miR-27a-3p, miR-27b-3p, and miR-124-3p on cell proliferation and gene expression in MEPM cells. (**A**) Cell proliferation assays using MEPM cells treated with inhibitors for control, miR-27a-3p, miR-27b-3p, or miR-124-3p. Statistical analysis was conducted by a two-way ANOVA with Dunnett's test (n=6). (**B-D**) Quantitative RT-PCR for the indicated genes after treatment with either control or miR-27a-3p inhibitor (B), either control or miR-27b-3p inhibitor (C), and either control or miR-124a-3p inhibitor (D). Statistical analysis was conducted by multiple *t*-tests adjusted by Bonferroni (n=3). *Adjusted $p < 0.00132$ in B (38 genes), adjusted $p < 0.00135$ in C (37 genes), adjusted $p < 0.000909$ in D (55 genes), **Adjusted $p < 0.000263$ in B (38 genes), adjusted $p < 0.000270$ in C (37 genes), adjusted $p < 0.000182$ in D (55 genes), ***Adjusted $p < 0.0000263$ in B (38 genes) vs control.

Supplementary Figure S2. Influence of *at*RA on cell proliferation and gene expression in MEPM cells. (**A**) Cell proliferation assays in MEPM cells treated with 10 or 30 µM *at*RA for 24, 48, and 72 hours. Statistical analysis was conducted by a two-way ANOVA with Dunnett's test (n=6). ***$p < 0.001$ vs control. NS, not significant. (**B**) Immunoblotting analysis for cleaved caspase 3 in MEPM cells treated with 30 µM *at*RA for 72 hours. The mouse intestine was used as a positive control (PC), and GAPDH was used as an internal control. Representative images from two independent experiments are shown. (**C**) Flow cytometry with APC Annexin-V/PI staining using MEPM cells treated with 30 µM *at*RA for 24 hours.

Supplementary Figure S3. Influence of *at*RA on gene expression in MEPM cells. Quantitative RT-PCR for the indicated genes (39 genes) after treatment with *at*RA for 24 hours in MEPM cells. Statistical analysis was conducted by multiple *t*-tests adjusted by Bonferroni (n=3). **Adjusted $p < 0.000256$ (39 genes), ***adjusted $p < 0.0000256$ (39 genes): downregulation compared to the control. $^{\#\#}$Adjusted $p < 0.000256$ (39 genes): upregulation compared to the control.

Supplementary Figure S4. Effect of miR-124-3p inhibitor on the expression of other miRs in MEPM cells. (**A**, **B**) Quantitative RT-PCR analyses for miR-27a-3p, miR-27b-3p, and miR-124-3p after treatment with either control or miR-124-3p inhibitor for 24 hours (A) and 48 hours (B). Statistical analysis was conducted by multiple *t*-tests adjusted by Bonferroni. ***Adjusted $p < 0.000333$ (3 miRs) vs control. ND, not detected.

Supplementary Table S1. Primer pairs used in this study.

**Gene**	**Forward primer**	**Reverse primer**
Acvr1	5'-AACATCCCACCAGAAACCCTC-3'	5'-GCTTCTCATCTTCCACACTCGG-3'
Acvr2a	5'-TACACCAGGAGGTTTGTCTCC-3'	5'-TACAATCAGTCCTGTCATAGCAGTT-3'
Adamts9	5'-TGCTTGGCAAAAGATGGAAGC-3'	5'-TGCTTGGCAAAAGATGGAAGC-3'
Alx1	5'-CCAGGGTCCAGGTTTGGTT-3'	5'-TTCAAATGCGTGTCCGTTGG-3'
Apaf1	5'-CCCCAGAGGCCGGTTATTTT-3'	5'-CAGAGACCTTGGGTGTTTGC-3'
Arid5b	5'-CCCTTGGCGACTTTTTCTTTGTA-3'	5'-TCTTCCCCGAGATCAGCTTTTT-3'
Axin1	5'-ACCTCACATTCCTCGCACTT-3'	5'-GTCTTAGCCACATGCCCACT-3'
Bmi1	5'-CGCTTGGCTCGCATTCATTTT-3'	5'-ATCTGACCTTATGTTCAGGAGTGG-3'
Bmp2	5'-GACCCGCTGTCTTCTAGTGT-3'	5'-ACGGCTTCTTCGTGATGGAA-3'
Bmpr1a	5'-CCCCTGTTGTTATAGGTCCGT-3'	5'-TTCACCACGCCATTTACCCA-3'
Cask	5'-TCACCCATGGCTTAAGGAGC-3'	5'-GAGAGACGGCTCTTTCTGCTG-3'
Cdc42	5'-ATGTGAAAGAAAAGTGGGTGCC-3'	5'-GATGCGTTCATAGCAGCACAC-3'
Chd7	5'-GTCCCGAGGCTCAGCAATAA-3'	5'-GTCCCGAGGCTCAGCAATAA-3'
Chrd	5'-CGACTGCTGCAAACAGTGTC-3'	5'-TCTCAGAGGACCTTTGGGCT-3'
Chuk	5'-GCACAGACCTCCTGATCACTT-3'	5'-GAGTTACCACACATGTCAGAGGA-3'

Csrnp1	5'-CCACCCAACATCTCTGGACC-3'	5'-GACGGGGGAGTGAAACTCTG-3'
Dicer1	5'-GATGGTTCTGGAAGGCCCG-3'	5'-CCTGGTTTGCAGAGTTGACG-3'
Dlx2	5'-TGGCTGATATGCACTCGACC-3'	5'-TGCTTTTGGCGGAGTAGGAG-3'
Dlx5	5'-TACTGCTCTCCTACCTCTGCTT-3'	5'-CAGATTTTCACCTGTGTTTGCGT-3'
Dnmt3b	5'-TGCTCAGACCTTGGAAACCTC-3'	5'-ATCTCCTGTCATGTCCTGCG-3'
Ednrb	5'-CCCTAAGGGTCTGCATGCTT-3'	5'-GCCACTTCTCGTCTCTGCTTT-3'
Efnb1	5'-TTGTGGCTATGGTCGTGCTG-3'	5'-CCCATTGGACGTTGATGTAATGTA-3'
Egfr	5'-ACGTCACATTGTTCGAAAGCG-3'	5'-TCACATAGGCTTCGTCAAGGAT-3'
Ephb2	5'-CGGTGTGATCCTGGACTACG-3'	5'-GCTCAAACCCCCGTCTGTT-3'
Esrp1	5'-GCCCCTCTGATTCCACTTCC-3'	5'-AGGGAGGAGACAGGCATGG-3'
Eya1	5'-GAGCTTGCGGCGAGGTAG-3'	5'-CATTGGCTCTGTTTTAACTGCAAC-3'
Eya4	5'-GCCGAAAGGGATGTCCTGT-3'	5'-GTCTTGCATTTCCATAGACCTGG-3'
Fbxw7	5'-AGCAGAGAAGATGAACATACGCA-3'	5'-CCGCTCCAGCTCTGAAACATT-3'
Fign	5'-CACGTTTCAAGGCAGTGACATT-3'	5'-CACTGCATCTTCAAGCCTCCT-3'
Fst	5'-TGCTACTCTGCCAGTTCATGG-3'	5'-CGTTCTCACACGTTTCTTTACAAGG-3'
Fzd7	5'-GGGGCGAGAGATGGTTTTGA-3'	5'-AGGCTACAGACAGAGCGGTA-3'
Gab1	5'-GAAGTGGTTTGCTCGGGATG-3'	5'-GCGGCTTCACAGGATCTTCTT-3'
Gabrb3	5'-GCCAGCATCGACATGGTTTC-3'	5'-CTGTCGTAGTGATCCTGAGCC-3'
Gas1	5'-CGGAGGAGCGTTGCTAATGT-3'	5'-ATACAAAAGTGGGCCGGGAG-3'
Glce	5'-GTATAAGTGGAGTTGAAGGTGTGC-3'	5'-GAACCACAGACACACTCCCAT-3'
Gli3	5'-TTCTGAGTCCTCACAGAGCAAG-3'	5'-CGTGAACAATCAAGCCAGCG-3'
Grb2	5'-TCAGTGGAATTAAAAAGGGTGGC-3'	5'-CTTGGCTCTGGGGATTTTGC-3'
Gsk3b	5'-AGCACCCTAACATAAAACAAAAGGA-3'	5'-GCTCTCGGTTCTTAAATCGCTTG-3'
Hic1	5'-CCCTAACGAAGGCCTGACA-3'	5'-TCCCGCCTCGAAAGAAGTTG-3'
Ift88	5'-TAGGATCAGGCGTCGCTTCT-3'	5'-GCAGTTACGGGAGGTCTTCT-3'
Insig2	5'-TTACCTGGAGAGAGTTCACCTTTT-3'	5'-AGGGGTACAACAGCCCAATC-3'
Itgb1	5'-CAAATGCCAAATCTTGCGGAGA-3'	5'-TTCTGTGGTTCTCCTGATCTCAAT-3'
Kif3a	5'-TGCCGTATCGGAACTCCAAA-3'	5'-CTTTCTTTTTACCTGCTTGGTCCC-3'
Lims1	5'-TGTGGAGCATTTTGTTTGTGCC-3'	5'-TTTGGCAACTCGCCTCTTAAAC-3'
Lrp6	5'-ATTATTGTCCCCGGATGGGC-3'	5'-ACTGCCTGCCGGTTTGTT-3'
Meox2	5'-TGGCAGCAAAAGGAAAAGCG-3'	5'-GGAACCACACTTTCACCTGTCT-3'
Mmp16	5'-AAACGGCAAACGTGATGTGG-3'	5'-GGATCTTGTCAGGTGGACCATA-3'
Nrp1	5'-AAGCGCAAGGCTAAGTCGTT-3'	5'-CTGTGGCCAGGACAGTGG-3'
Pax3	5'-TACAGACAGCTTTGTGCCTCC-3'	5'-AGATAATGAAAGGCACTTTGTCCAT-3'
Pax9	5'-ACCGCTTCTGCACTCTGATG-3'	5'-GGGCAACACAAATGCCTCAT-3'
Pbx3	5'-CATCGGCGACATCCTCCAC-3'	5'-TGTGAATTCATTACATGCCTGTTCA-3'
Pdgfra	5'-TCAGAGAGAATCGGCCCCA-3'	5'-AGGACGAATTCAGCTGCACA-3'
Pds5b	5'-ACAAGGACCAACGATGGGAAAA-3'	5'-AACCCATGCAATGTTCTCAAGTAA-3'

Pkdcc	5'-CCTCTACAATGCCTACAGGTTCTT-3'	5'-TGAGCATGGCTCTCACAGAC-3'
Prdm16	5'-TGCGATCCAAGGCGAGG-3'	5'-TATCCGTCAGCATCTCCCATC-3'
Prrx1	5'-AGCAGGACAATGACCAGTTGAA-3'	5'-CCAGTCATTCACCTGTACGGA-3'
Ptpn11	5'-GGTGAATGACTTCTGGCGGC-3'	5'-CTCTCTGTGTTTCCTTGTCCGA-3'
Rac1	5'-TTGGTAAAACCTGCCTGCTCA-3'	5'-AAGAACACGTCTGTCTGCGG-3'
Ror2	5'-TGAAGAATGATGCCCCGGTT-3'	5'-AAGGCAGCTGTGATTCGGTTT-3'
Runx1	5'-GCTTTCGCAGAGCGGTGA-3'	5'-ACCTTGAAAGCGATGGGCAG-3'
Satb2	5'-CCTCAAAATCACACACCAGCA-3'	5'-GGGACCTTGGTGTTGGAACTA-3'
Sgpl1	5'-CCGAGGCGCGTGAGAC-3'	5'-AGACCATAAACTCTCTGGCTGG-3'
Six1	5'-AAGCAGGTCATCGGAAGCTC-3'	5'-ATGAGCAAGCCAACCCTGTT-3'
Six4	5'-CCCCACCGGGCAGATTG-3'	5'-AAGTTCCGAGTGGAGTTGTACC-3'
Ski	5'-GTTGTACACTCGCACAAGGC-3'	5'-CGAGGCTCTTATTGGAGGAACC-3'
Smad4	5'-CTCTCAGGATTAACACTGCAGAG-3'	5'-GTTGATGCGCGATTACTTGGC-3'
Snai2	5'-GAAGCCCAACTACAGCGAAC-3'	5'-ATAGGGCTGTATGCTCCCGA-3'
Sos1	5'-GCAGCACTTTATTTGCAGTCCA-3'	5'-TGATGGCTAGCTGTTTCCCC-3'
Sox11	5'-CAGCGAGAAGATCCCGTTCA-3'	5'-GGGTCCGTCTTGGGCTTTT-3'
Sox9	5'-AGCACAAGAAAGACCACCCC-3'	5'-ATGTGAGTCTGTTCCGTGGC-3'
Sp8	5'-TTAAACTTGACTCCGCCG CT-3'	5'-AGCAAAACTAGGCCCGGAAA-3'
Spry1	5'-CACTAGCCGGCGTGGC-3'	5'-TCTCCAGTTCCAGCAGTCAG-3'
Spry2	5'-ACCGATTGCTTGGAAGTTGGA-3'	5'-TTGGTGTTTCGGATGGCTCT-3'
Sumo1	5'-ATGTCTGACCAGGAGGCAAAA-3'	5'-TTATCTAAACCGTCGAGTGACCC-3'
Tgfbr1	5'-GGCCGGGCCACAAACA-3'	5'-CTGAAAAAGGTCCTGTAGTTGGG-3'
Tgfbr3	5'-TCTTCCTGGTTTCGGAGGGT-3'	5'-AGGATTGGAGTTGGGGGAGA-3'
Tm7sf2	5'-TTTGCGACCACTCTCACCAG-3'	5'-AGGACAGACTCCTCATACCAGA-3'
Tshz1	5'-CGCTCGGCAGCTTACGTT-3'	5'-GATCTGTGCCAAGCTGTCCT-3'
Vcan	5'-ATGACTTCCGCTGGACTGAC-3'	5'-GTATGCAGATGGGTTCATGCAG-3'
Zeb1	5'-GGAGGTGACTCGAGCATTTAGA-3'	5'-ACTCGTTGTCTTTCACGTTGTC-3'
Gapdh	5'-AACTTTGGCATTGTGGAAGG-3'	5'-ACACATTGGGGGTAGGAACA-3'

Supplementary Table S2. Target genes of each candidate microRNA

**miRNA**	**Target genes**
miR-27a-3p	*Acvr1*, *Acvr2a*, *Apaf1*, *Bmi1*, *Bmp2*, *Bmpr1a*, *Cask*, *Cdc42*, *Chd7*, *Csrnp1*, *Dicer1*, *Egfr*, *Ephb2*, *Eya1*, *Eya4*, *Fbxw7*, *Fzd7*, *Gab1*, *Gabrb3*, *Gsk3b*, *Hic1*, *Mmp16*, *Pax3*, *Pax9*, *Pdgfra*, *Pds5b*, *Prdm16*, *Runx1*, *Satb2*, *Six1*, *Sos1*, *Sox11*, *Spry1*, *Spry2*, *Sumo1*, *Tgfbr1*, *Tgfbr3*, *Zeb1*
miR-27b-3p	*Acvr1*, *Acvr2a*, *Apaf1*, *Bmi1*, *Bmp2*, *Cask*, *Cdc42*, *Chd7*, *Csmp1*, *Dicer1*, *Egfr*, *Ephb2*, *Eya1*, *Eya4*, *Fzd7*, *Gab1*, *Gabrb3*, *Gsk3b*, *Hic1*, *Mmp16*, *Pax3*, *Pax9*, *Pdgfra*, *Pds5d*, *Prdm16*, *Runx1*, *Satb2*, *Six1*, *Smad4*, *Sos1*, *Sox11*, *Spry1*, *Spry2*, *Sumo1*, *Tgfbr1*, *Tgfbr3*, *Zeb1*
miR-124-3p	*Adamts9*, *Alx1*, *Arid5b*, *Axin1*, *Bmpr1a*, *Cask*, *Cdc42*, *Chrd*, *Chuk*, *Dlx2*, *Dlx5*, *Dnmt3b*, *Ednrb*, *Efnb1*, *Esrp1*, *Eya1*, *Eya4*, *Fign*, *Fst*, *Gas1*, *Glce*, *Gli3*, *Grb2*, *Gsk3b*, *Hic1*, *Ift88*, *Insig2*, *Itgb1*, *Kif3a*, *Lims1*, *Lrp6*, *Meox2*, *Mmp16*, *Nrp1*, *Pax3*, *Pbx3*, *Pkdcc*, *Prrx1*, *Ptpn11*, *Rac1*, *Ror2*, *Sgpl1*, *Six4*, *Ski*, *Snai2*, *Sos1*, *Sox9*, *Sp8*, *Spry1*, *Tgfbr1*, *Tgfbr3*, *Tm7sf2*, *Tshz1*, *Vcan*, *Zeb1*

Figure S1. Yoshioka et al.

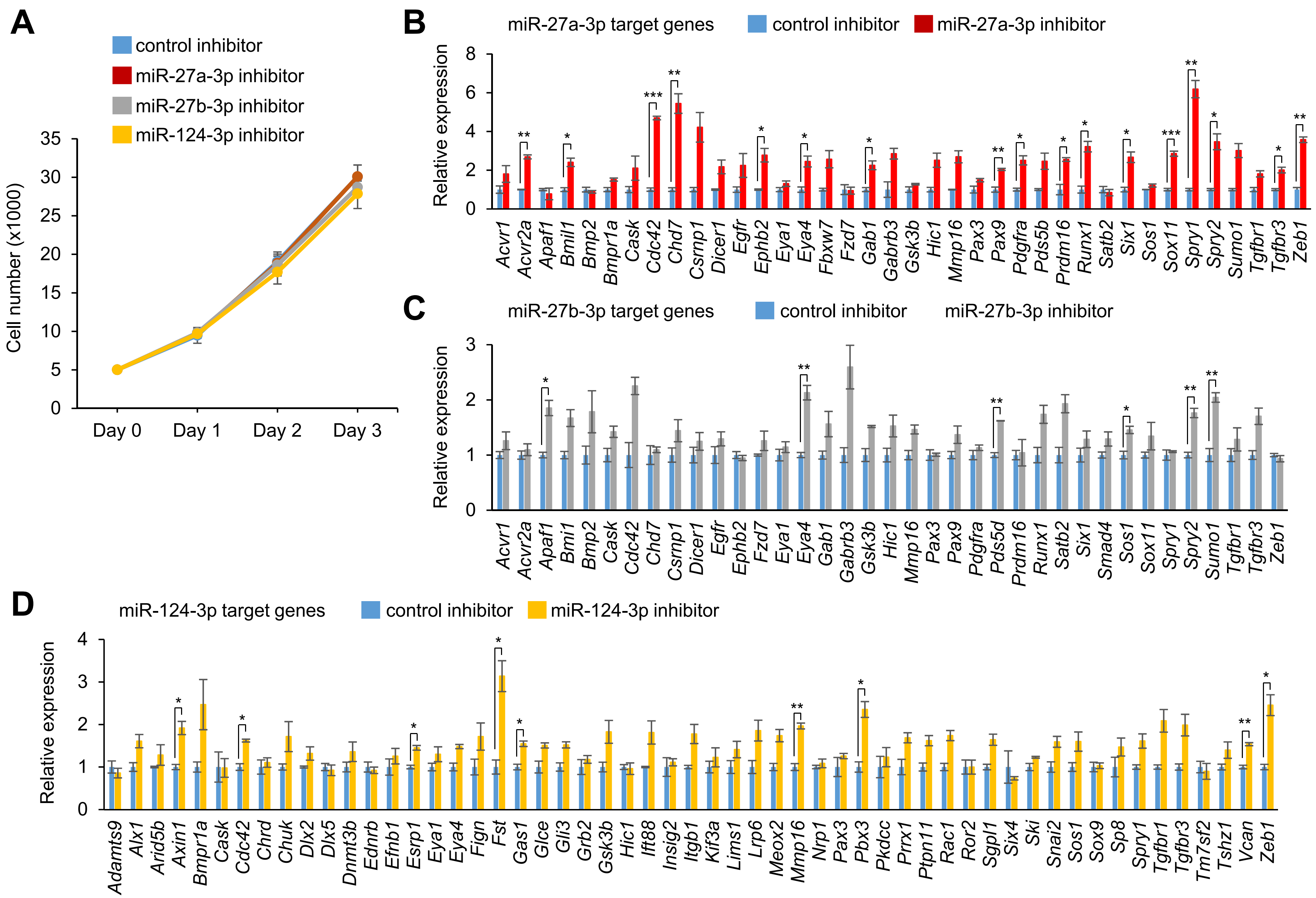

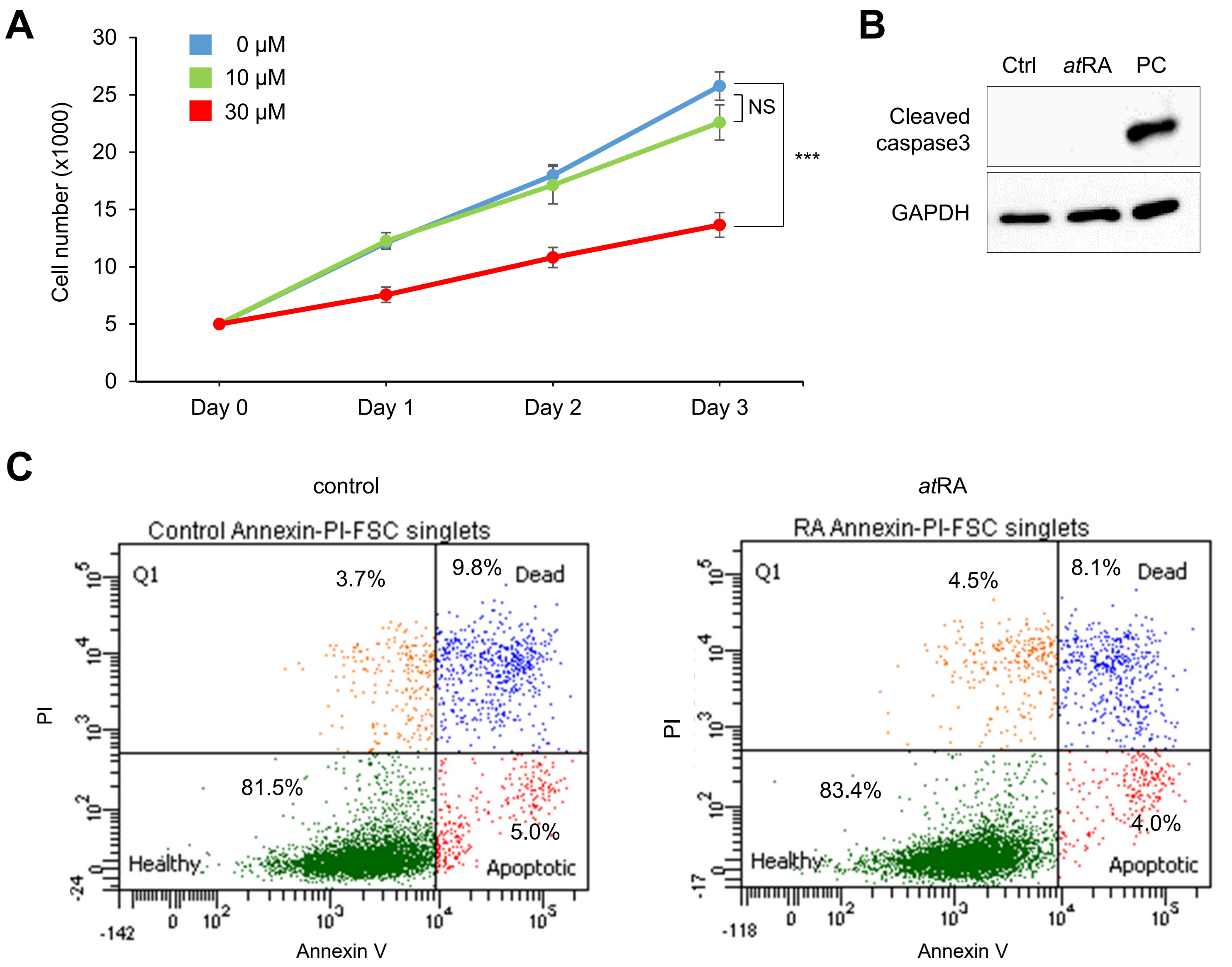
A
0 μM
10 μM
30 μM
Cell number (x1000)
30
25
20
15
10
5
0
Day 0
Day 1
Day 2
Day 3
NS
***
B
Ctrl
atRA
PC
Cleaved caspase3
GAPDH
C
control
atRA
Control Annexin-PI-FSC singlets
RA Annexin-PI-FSC singlets
Q1
3.7%
9.8%
Dead
81.5%
5.0%
Healthy
Apoptotic
4.5%
8.1%
83.4%
4.0%
PI
Annexin V

Figure S3. Yoshioka et al.

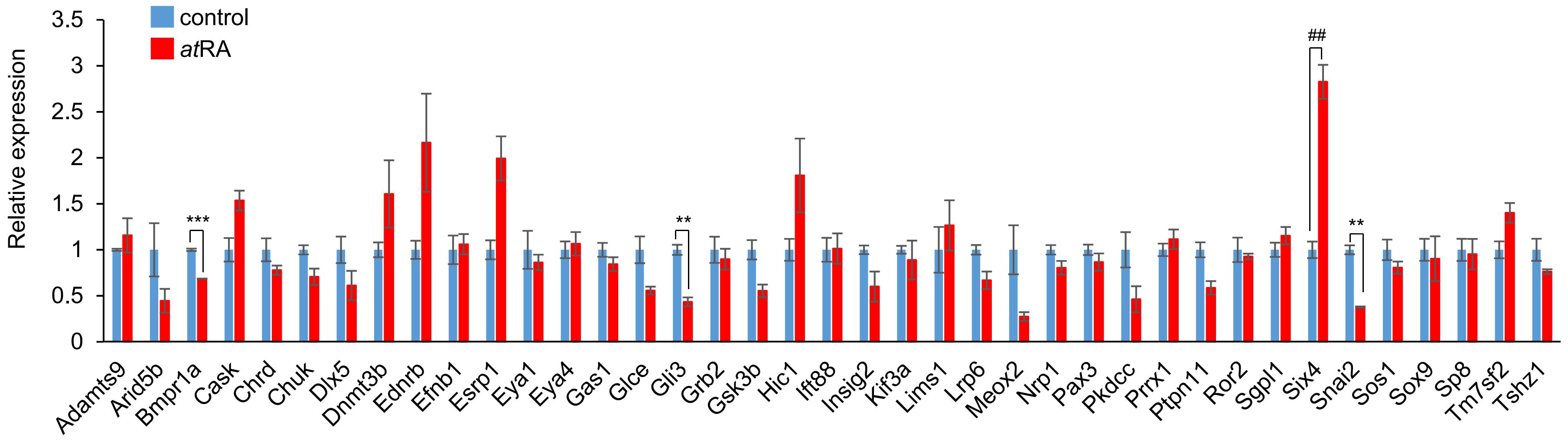

Figure S4. Yoshioka et al.

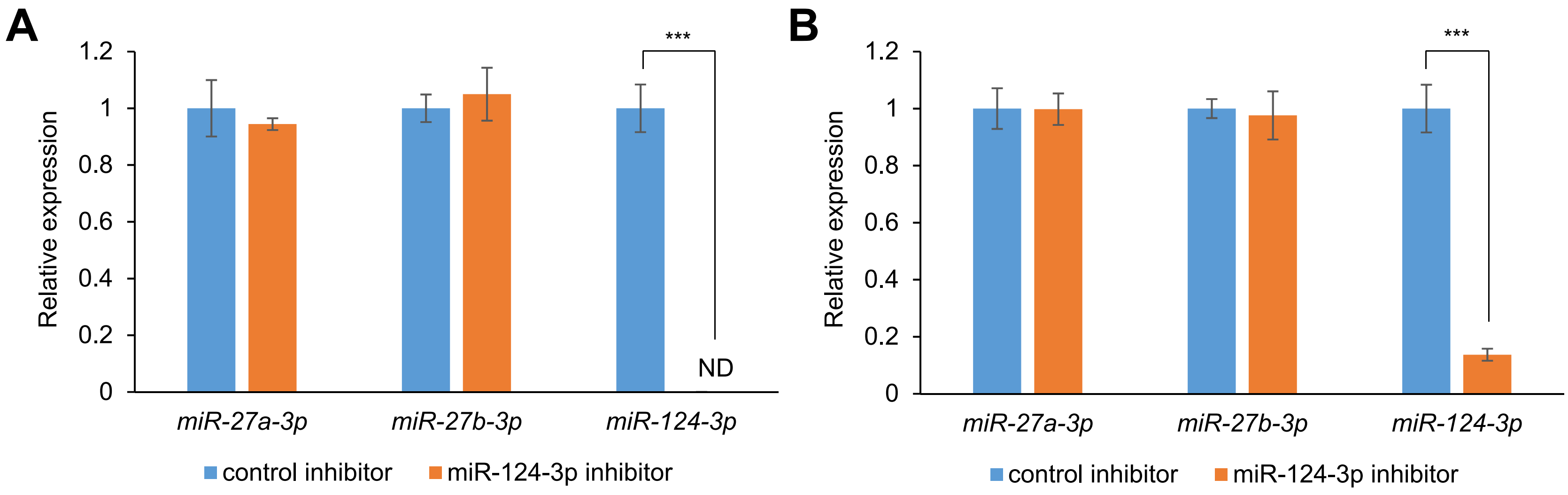

Supplement: Supplementary file 1 [file Data_Sheet_1.PDF]
